# Herpes zoster and long-term vascular risk: a retrospective cohort study

**DOI:** 10.1038/s41598-023-29667-w

**Published:** 2023-02-09

**Authors:** Amir Horev, Anat Horev, Adi Gordon-Irshai, Michal Gordon, Nicolas Andre, Gal Ifergane

**Affiliations:** 1 Pediatric Dermatology Service, Soroka University Medical Center, POB 151, 84101 Beer Sheva, Israel; 2 Faculty of Health Sciences, Ben-Gurion University of the Negev, Beer Sheva, Israel; 3 Neurology Department, Soroka University Medical Center, Beer Sheva, Israel; 4 Clinical Research Center, Soroka University Medical Center, Beer-Sheva, Israel

**Keywords:** Infection, Cardiovascular diseases, Infectious diseases, Skin diseases

## Abstract

Herpes zoster (HZ) represents a serious health problem in the general population due to its abundance and complications. Stroke and acute myocardial infarction are well-documented short-term complications of HZ, primarily due to vasculopathy in the cerebral and coronary arteries. However, no major study to date has specifically demonstrated that HZ is a long-term risk factor for all Major Adverse Cardiac and Cerebrovascular Events (MACCE). A retrospective cohort study was conducted analyzing the association between HZ and MACCE. We compared HZ patients diagnosed between 2001 and 2018 and a matched control group. The model was stratified according to matched pairs and adjusted for age, socioeconomic status, history of dyslipidemia, and prior myocardial infarction (MI). Association between HZ exposure and stroke was assessed through a multivariable Cox regression analysis. The study included 41,930 patients, with 20,965 patients in each group. The risk of MACCE was 19% higher among HZ patients in the first year of follow up (P < 0.001). Antiviral treatment did not positively affect long-term survival among HZ patients (P < 0.001). These results suggest that HZ is a marker of long-term vascular risk. Additional studies will be needed to further evaluate this risk, the impact of HZ vaccination on such risk, and potential mitigation strategies.

## Introduction

Herpes zoster (HZ) infections are characterized by painful, unilateral vesicular skin eruptions usually limited to a single dermatome. These infections are caused by the endogenous reactivation of latent varicella-zoster virus (VZV) residing in the sensory ganglia and dorsal roots after primary infection. HZ represents a serious health problem in the general population^1^, with an overall lifetime risk range from 10 to 30%^2^. Postherpetic neuralgia, the most debilitating HZ complication, affects 13–25% of patients^3^. It often negatively affects the quality of life and sometimes requires specific treatment^4^. Another complication of this infection is encephalitis due to the viral dissemination to the central nervous system, which may lead to a viral-induced, cerebral angiitis^5^. Viral DNA can be detected in the cerebrospinal fluid (CSF) during an acute HZ event, provoking concern regarding the potential cerebrovascular implications of HZ infections. This concern and related clinical observations have spurred several studies and meta-analyses that have evaluated the cerebrovascular risk following HZ events and demonstrated increased short-term (weeks to months) stroke risk following HZ^6^. It has been suggested that vasculopathy caused by a productive VZV infection in the cerebral arteries results in pathological vascular remodeling and, subsequently, stroke^7^. However, it is unclear whether this increased stroke risk persists for years and whether it is confined to the cerebrovascular system or represents a more general form of vascular risk that includes the cardiovascular system. In this study, we sought to estimate the relationship between exposure to HZ and the long-term risk of major adverse cardiac and cerebrovascular events (MACCE) and to evaluate the impact of antiviral therapy on this relationship.

## Methods

The study was designed as a retrospective cohort study. Subjects with HZ were compared to those without HZ with respect to their cerebrovascular and cardiovascular morbidity during the study follow-up period.

### Study population

Our study population was defined as all adult (> 18 years old) residents of southern Israel insured by "Clalit", an Health Maintenance Organization (HMO) covering approximately 70% of the local population. As healthcare is universal in Israel, with “Clalit” being the largest non-for profit HMOs accessible to all, our study group is representative of the entire population of our region. The subjects in this area would be hospitalized in the Soroka University Medical Center (SUMC), a 1200-bed local hospital, as this is the only facility providing tertiary services to the metropolitan area of Beer-Sheva and the southern Israel region, home to up to 1 million residents. Subjects diagnosed with HZ identified based on ICD9 codes (053) or having a positive lab result for HZ (PCR, biopsy, Tzanck smear, or serology results) between Jan 2001 and Dec 2018 were included in the HZ-exposed group. Diagnoses were made by the patient's primary physician, dermatologist, or neurologist. Subjects without HZ in their medical history were matched to subjects in the HZ group at a 1:1 ratio based on follow-up time, starting for non-HZ subjects on the HZ diagnosis date of their matched subjects. We extracted all relevant demographic and medical history information recorded by the Clalit HMO primary care physicians and the Admission-Discharge-Transfer (ADT) hospital system of SUMC. Patient diagnoses were identified using ICD9 codes.

### Study endpoints

The study's primary outcome was MACCE incidence, which included stroke, Transient Ischemic Attack (TIA), Acute Myocardial Infarction (AMI), Percutaneous Coronary Intervention (PCI), Coronary Artery Bypass Grafting (CABG) incidence, and all cause death. Stroke or AMI events were defined as secondary outcomes. Patient diagnoses were identified using ICD9 codes (stroke 430–437; TIA V12.54; AMI 410–414). The endpoints were estimated at the maximum follow-up interval of 18 years.

### Statistical analysis

Descriptive statistics were presented for the entire study population and stratified according to HZ-exposure status. Continuous variables were expressed as means ± standard deviation (SD), medians, and minimum/maximum values. Categorical variables were presented as frequencies and percentages out of the available cases. Between-group comparisons of baseline covariates were performed using t-tests or Wilcoxon tests for continuous variables and chi-square tests or Fisher’s exact test for categorical variables. Cumulative event distributions of the endpoints were presented using the Kaplan–Meier method, and was calculated for the entire study period (up to 17 years). Time to events was compared among subgroups with the log-rank test. Multivariable Cox regression analyses were conducted to assess the relationship between HZ exposure and stroke, MI, and MACCE incidence over three follow-up periods (1 year, 5 years, and 15 years). Regression analyses were conducted incorporating several variables of clinical importance, and those variables exhibiting statistical significance were included in the final model. Cox regression analyses were stratified according to matched pairs and adjusted for socioeconomic status, age, history of dyslipidemia, and prior AMI. Given the relatively wide caliper (± 5 years) used for age matching, the age adjustment of this regression model was imperative to avoid any remaining confounding effects of age on the resultant data. Socioeconomic status, the history of dyslipidemia, and prior AMI also differed significantly between groups and were regarded as possible confounders. Proportional hazard assumption was tested by inspection of survival curves for categorical covariates and tested based on Schoenfeld residuals for the continuous covariates. A two-tailed P < 0.05 was the significance threshold for this statistical testing. SPSS 26.0 was used for all data analyses. Soroka Medical Center’s institutional review board (IRB) approved the study, which complied with the 1964, 1975 and 2013 revisions of the Helsinki Declaration. Due to the retrospective nature of the study, Soroka Medical Center’s IRB waived the need of obtaining informed consent.

## Results

Overall, 25,209 subjects were diagnosed with HZ during the study period, of whom 4244 were excluded due to a lack of matching subjects from the non-HZ group. In all, 41,930 patients were included in this study (20,965 per group). The average age at zoster diagnosis was 45.7 (± 18.0) years. Of these patients, 33.8 were male, 18.5% had diabetes mellitus, and 29.9% had hypertension as underlying diagnoses. The baseline characteristics of these participants are shown in Table 1. The follow-up period did not differ significantly between the study groups, with a minimum of 0 and a maximum of 17 years, and a mean follow-up interval of 4.4 years. Cumulative MACCE survival at the end of the follow-up period was 54.4% (1970 events) in the HZ group as compared to 74.2% (1774 events) in the non-HZ group (P < 0.001). The stroke cumulative survival rate was 88.7% (364 events) in the HZ group as compared to 94.0% (318 events) in the non-HZ group (P = 0.062). The AMI cumulative survival rate was 68.7% (988 events) in the HZ group and 90.0% (823 events) in the non-HZ group (P < 0.001) (Table 2).

**Table 1:** Clinical and demographic characteristics of study population grouped according to Zoster exposure status.

Subject's characteristic	Non-HZ group N = 20,965	HZ group N = 20,965	P-value
Males, % (n/N)	33.8 (7081/20,965)	33.8 (7081/20,965)	
Age at zoster, years			0.847
Mean ± SD (n)	45.7 ± 18.0 (n = 20,965)	45.7 ± 18.1 (n = 20,965)	
Median	43.0	43.0	
Min; max	18; 88	18; 88	
Diabetes mellitus, % (n/N)	18.5% (3883/20,965)	18.5% (3883/20,965)	
Hypertension, % (n/N)	29.9 (6270/20,965)	29.9 (6270/20,965)	
Dyslipidemia, % (n/N)	7.7 (1612/20,965)	8.7 (1829/20,965)	< 0.001
Prior stroke, % (n/N)	0.4 (77/20,965)	0.3 (70/20,965)	0.563
Prior MI, % (n/N)	2.3 (474/20,965)	2.9 (617/20,965)	< 0.001
Smoking, % (n/N)	21.7 (2223/20,965)	21.5 (2069/20,965	0.637

**Table 2:** Post zoster vascular event survival, throughout the entire study period.

Parameter	Non-Zoster	Zoster	P-value
MACCE, survival %* ± SD (n)	74.2% ± 0.02 (1774)	54.4% ± 0.13 (1970)	< 0.001
Stroke, survival %* ± SD (n)	94.0% ± 0.02 (318)	88.7% ± 0.03 (364)	0.062
Acute MI, survival %* ± SD (n)	90.0% ± 0.02 (823)	68.7% ± 0.16 (988)	< 0.001
Stroke or MI, survival %* ± SD (n)	84.3% ± 0.03 (1033)	62.4% ± 0.14 (1213)	< 0.001

*Multivariable regression is adjusted to gender, age, diabetes, hypertension, socioeconomic status, dyslipidemia, and prior AMI.

**Figure 1:**
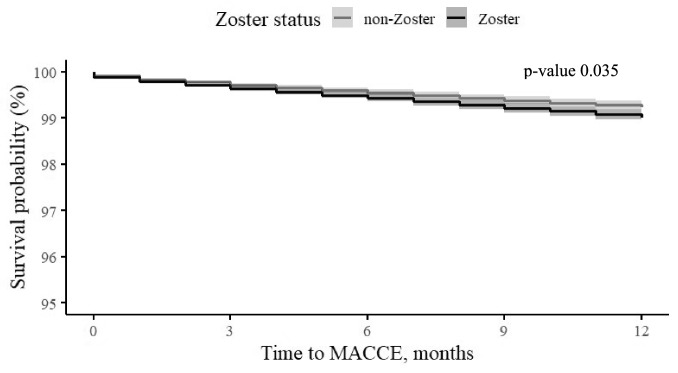
Adjusted* MACCE-free survival time at the first year after exposure to zoster, by zoster status (95% CI). *Adjusted to gender, age, socioeconomic status, diabetes mellitus, hypertension, socioeconomic status, dyslipidemia and prior AMI.

**Figure 2:**
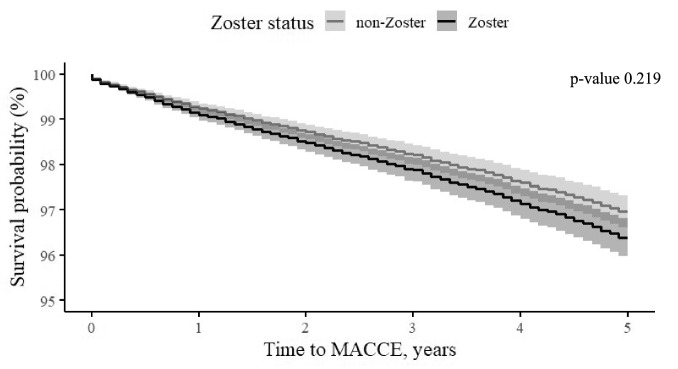
Adjusted* MACCE-free survival time 5-years after exposure to zoster, by zoster status (95% CI). *Adjusted to gender, age, socioeconomic status, diabetes mellitus, hypertension, socioeconomic status, dyslipidemia and prior AMI.

**Figure 3:**
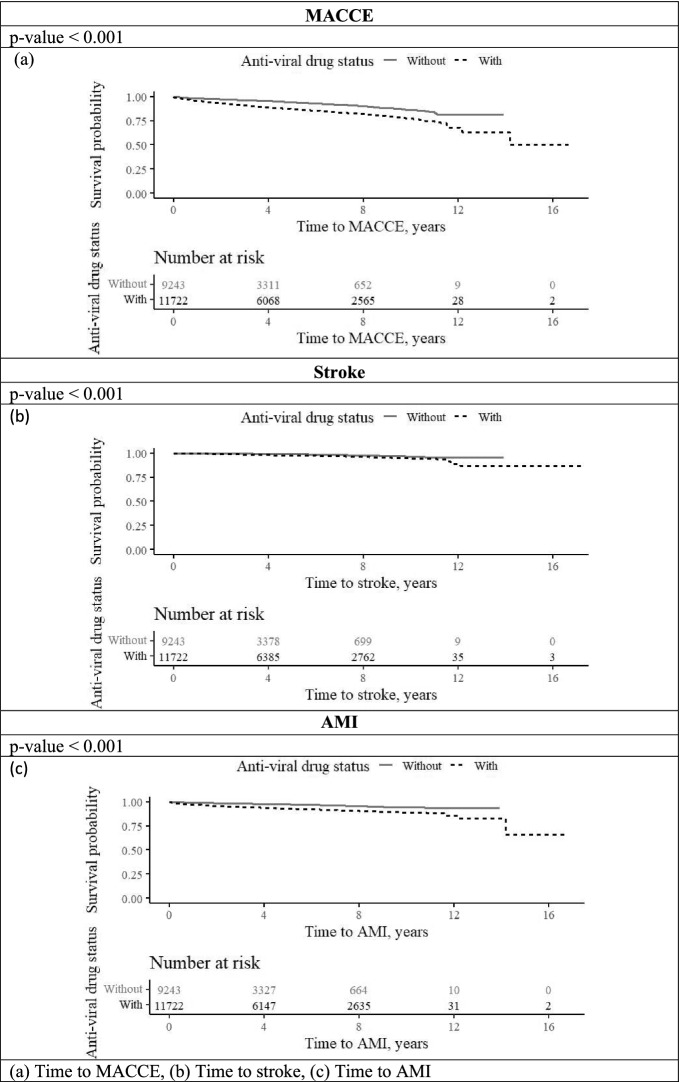
Event-free survival in zoster subjects, by use of antiviral drug one month after exposer to zoster.

In a multivariable analysis adjusted for age, socioeconomic status, dyslipidemia, and prior AMI, we estimated the hazard ratio (HR) associated with prior HZ exposure compared to non-HZ subjects after 1, 5, and 15 years following exposure. The hazard ratio determines whether a patient in the HZ group at any given time has a greater, equal, or lower probability of experiencing MACCE during the next unit of time than a subject in the non-HZ group. As such, any value above 1, will demonstrate a higher probability of experiencing MACCE. The HR for MACCE incidence in the HZ group was 1.19 (95% CI 1.01; 1.39, P = 0.035) after 1 year, 1.07 (95% CI 0.96; 1.18, P = 0.219) after 5 years, and 1.03 (95% CI 0.95; 1.13, P = 0.451) after 15 years (Table 3). Analyses of the MACCE-free interval revealed a significant difference in survival between the HZ group and the non-HZ in the year following HZ exposure (Fig. 1), but the difference was not significant after 5 years (Fig. 2). Analyses of the stroke-free interval and MI-free interval can be found in the appendix, represented by Figure A and B. Analyses of stroke-free, MI-free, and MACCE-free survival were conducted to assess the potential impact of treatment with antiviral drugs within a month after exposer to zoster (acyclovir, famciclovir, and valaciclovir) on HZ patients. The analyses revealed lower survival rates in subjects treated with antiviral medications (Fig. 3).

**Table 3:** Multivariable Cox regression for HZ exposure on the study outcomes.

	Years from exposure to zoster		
	1	5	15
MACCE			
Hazard ratio (95% CI)	1.19 (1.01; 1.39)	1.07 (0.96; 1.18)	1.03 (0.95; 1.13)
P value	0.035	0.219	0.451
Stroke			
Hazard ratio (95% CI)	0.78 (0.50; 1.21)	1.04 (0.83; 1.31)	1.13 (0.93; 1.37)
P value	0.271	0.748	0.208
AMI			
Hazard ratio (95% CI)	1.30 (0.95; 1.78)	1.07 (0.87; 1.33)	1.02 (0.85; 1.22)
P value	0.098	0.486	0.867

The HR values corresponding to stroke and AMI risk when comparing HZ and non-HZ subjects were estimated in a multivariable analysis adjusted for age, socioeconomic status, dyslipidemia, and prior AMI. For stroke the estimated HR was 0.78 (95% CI 0.50; 1.21, P = 0.271) after 1 year, 1.03 (95% CI 0.82; 1.30, P = 0.791) after 5 years, and 1.12 (95% CI 0.93; 1.36, P = 0.226) after 15 years. With respect to AMI the estimated HR was 1.30 (95% CI 0.95; 1.78, P = 0.098) after 1 year, 1.07 (95% CI 0.87; 1.33, P = 0.486) after 5 years, and 1.02 (95% CI 0.85; 1.22, P = 0.867) after 15 years (Table 3).

## Discussion

In the current analysis, we investigated whether suffering from an acute episode of HZ is associated with increased long-term vascular risk using an extensive HMO computerized database. Though they failed to reach significance after 5 years of follow up, our results nonetheless show that HZ infection trended towards a long-term increased risk of ischemic events in our cohort, including both cerebro vascular and coronary events. The risk of MACCE was 19% higher among HZ sufferers in the first year of follow up, and this risk was sustained for at least 4.4 years following the episode. It was not affected by the administration of antiviral agents during the HZ episode.

Previous studies have primarily reported increased short-term (weeks to months) stroke risk after an HZ episode. Schink et al.^8^ demonstrated that stroke risk increased in the first week following HZ infection and then fell over the subsequent 6–12-month follow-up period. Minassiant et al.^9^ observed an increase in stroke incidence a few weeks after HZ infection and a gradual decline in the risk of stroke in the following weeks. Sreenivasan et al.^10^ reported a peak in stroke incidence 2 weeks following HZ infection when analyzing a vast database (4.6 million enrollees) in Denmark followed by the moderation of this risk over a 1-year period. A similar pattern of increased risk of stroke in the following weeks after HZ infection and then declining risk during the following months was also demonstrated by Langan et al.^11^. A meta-analysis conducted by Liu et al.^12^ summarizing data from eight studies likewise showed a short-term increase in stroke risk followed by a decline in such risk following HZ infection, with the highest level of risk during the first 2 weeks (risk ratio [RR]: 2.36), with the RR falling to 1.56, 1.17, and 1.03 at 1, 3, and 6 months, respectively.

Interestingly, Marra et al.^13^ found no association between HZ and stroke over a long 3-year follow-up interval. Early post-HZ stroke and AMI risk has been attributed to a hypercoagulable state caused by prothrombotic autoimmune antibodies such as anticardiolipin forming during the HZ infection^14^, circulating immune complexes, and systemic inflammation^15^.

In their retrospective study, Breuer et al. showed that the HR for TIA and MI but not stroke were increased in all patients with HZ^16^. However, HZ patients included in this study had significantly more cardiovascular risk factors (diabetes, hypercholesterolemia, hypertension, smoking) than matched control subjects, which accounted for a major bias in the long term follow-up of vascular events in HZ subjects. This can also be said of the Danish study aforementioned though both studies identified the risk of stroke and TIA to be highest in those whose HZ occurred under the age of 40 years^10,16^. While our study did not segregate age groups, our robust adjustment method mitigated this bias and gave us a more neutral analysis of the long term effects of HZ on all major adverse cardiac and cardiovascular events, including stroke, TIA, MI.

In their propspective study, Curhan et al. demonstrated similar results to our observations and showed a long term implication of HZ in stroke and coronary heart disease^17^. Though they based their analysis off of self reported information on HZ without considering treatment, our findings expand on their results, and together imply that the increased risk is not limited to the cerebrovascular system but instead represents an elevated level of systemic cerebrovascular and coronary risk^16^. It is difficult to determine from our results whether this increased risk is caused by the HZ event, potentially resulting from inflammatory and prothrombotic changes that may persist for years, or whether the HZ episode is instead a marker for increased vascular risk, with patients exhibiting greater vascular risk when being exposed to HZ.

Though former studies on the short-term^7,11,18,19^ and long-term^10^ cardiovascular outcomes after antiviral use in HZ patients exhibited significant positive effects, our results tend to show the opposite effect, even suggesting a negative outcome of antiviral treatments on long-term survival. This should be put in perspective as our study design did not allow us to review detailed information regarding treatment options, treatment plans, or patients' therapeutic compliance over a 15-year follow-up period. However, we should also consider that in clinical practice, antiviral agents are more frequently given to HZ patients with underlying comorbid conditions. Though they received treatment in the acute phase of the infection, the long-term residual effects of HZ may have negatively impacted already fragile vascular homeostasis in these individuals, further decreasing long-term survival. Therefore, we believe that it is more reasonable to interpret HZ as a marker of high vascular risk rather than a causative factor.

The current study has some limitations. First, the case definition was based on administratively collected data, leaving cases of undiagnosed HZ patients unrecorded in the database. Such misclassification can potentially decrease the effect size toward the null hypothesis. Second, our data did not include HZ vaccination status, which could be a limitation given that recent literature has shown a decreased risk of neurologic post-infection sequelae in vaccinated patients^20^. Third, because our study focused mainly on the impact of HZ on vascular effects and did not comprise detailed information on vaccinations and treatments, only limited data regarding the long-term influence of HZ therapeutics on cardiovascular pathologies could be collected. Lastly, missing clinical details, such as HZ dermatome location, precluded any in-depth analysis of the exposure type or its implications. However, our study has some noteworthy strengths. This is the first study to explore the risk of cardiovascular events in HZ patients compared with non-HZ patients over a 15-year period. In addition, the unique setup of a centralized healthcare system with a single tertiary hospital treating all the acute patients in the region allows for the reliable evaluation of vascular risk in a very large cohort of patients.

The findings of our study suggest that HZ is a long-term vascular risk marker. Based on our observation, clinicians should consider reevaluating the vascular risk profile of patients recovering from HZ. Further studies are warranted to determine how a history of HZ should be incorporated into cardiovascular risk calculators and to evaluate the long-term impact of vaccines and mitigation strategies on such risk.

## Supplementary Information

Supplementary Information.

## Data Availability

The datasets generated during and/or analysed during the current study are not publicly available due privacy and IRB restrictions but are available from the corresponding author on reasonable request.

## Abbreviations

ADT: Admission-discharge-transfer
AMI: Acute myocardial infarction
CABG: Coronary artery bypass grafting
CI: Confidence interval
DNA: Deoxynucleic acid
HMO: Health maintenance organization
HR: Hazard ratio
HZ: Herpes zoster
ICD9: International Classification of Diseases, Ninth Revision
MACCE: Major adverse cardiac and cerebrovascular events
MI: Myocardial infarction
PCI: Percutaneous coronary intervention
PCR: Polychain reaction
SUMC: Soroka University Medical Center
TIA: Transient ischemic attack
VZV: Varicella-zoster virus
