# Supplementary material for: Herpes zoster and long-term vascular risk: a retrospective cohort study
Source: Sci Rep. 2023 Feb 9;13:2364. doi: 10.1038/s41598-023-29667-w (PMC9911591; doi:10.1038/s41598-023-29667-w)

Herpes Zoster and Long-Term Vascular Risk: A Retrospective Cohort Study

*Amir Horev[1,2], *Anat Horev[2,3], Adi Gordon Irshai[2,4], Michal Gordon[4], Nicolas Andre[2], Gal Ifergane[2,3]

[1]Pediatric Dermatology Service, Soroka University Medical Center, Beer Sheva, Israel

[2]Faculty of Health Sciences, Ben-Gurion University of the Negev, Beer Sheva, Israel

[3]Neurology Department, Soroka University Medical Center, Beer Sheva, Israel

[4]Clinical Research Center, Soroka University Medical Center, Beer-Sheva, Israel

*Equal contributors

Corresponding author:

Amir Horev, MD

Soroka University Medical Center

POB 151, Beer Sheva, 84101, Israel

Email: amirhr@clalit.org.il

ORCID ID: 0000-0001-6646-9061

Appendix

Figure A: Adjusted* Stroke-free survival time throughout the entire study period, by zoster stat us (95% CI)

*Adjusted to gender, age, socioeconomic status, diabetes mellitus, hypertension, socioeconomic status, dyslipidemia and prior AMI

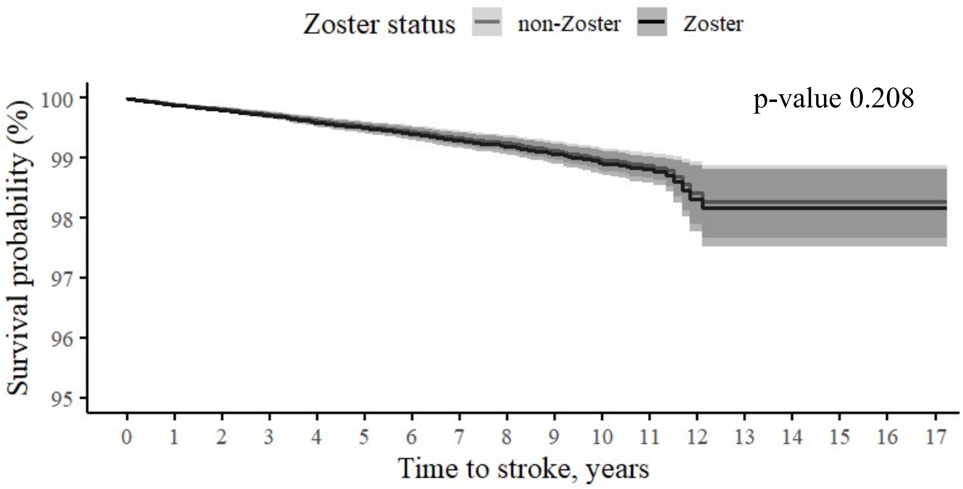

Figure B: **Adjusted* AMI-free survival time throughout the entire study period, by zoster status (95% CI)**

*Adjusted to gender, age, socioeconomic status, diabetes mellitus, hypertension, socioeconomic status, dyslipidemia and ure B: AMI

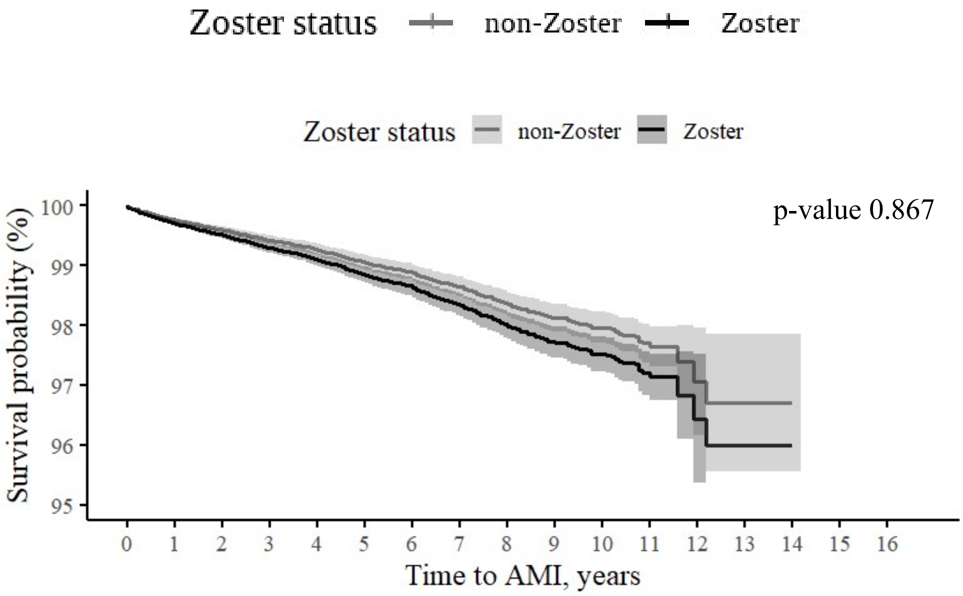

Supplement: Supplementary file 1 — Supplementary Information. [file 41598_2023_29667_MOESM1_ESM.pdf]
